# Tea and Chicory Extract Characterization, Classification and Authentication by Non-Targeted HPLC-UV-FLD Fingerprinting and Chemometrics

**DOI:** 10.3390/foods10122935

**Published:** 2021-11-28

**Authors:** Josep Pons, Àlex Bedmar, Nerea Núñez, Javier Saurina, Oscar Núñez

**Affiliations:** 1 Department of Chemical Engineering and Analytical Chemistry, University of Barcelona, Martí i Franquès 1-11, E08028 Barcelona, Spain; josepferre8@gmail.com (J.P.); alexbedmar1999@gmail.com (À.B.); nereant7@gmail.com (N.N.); xavi.saurina@ub.edu (J.S.); 2 Research Institute in Food Nutrition and Food Safety, University of Barcelona, Recinte Torribera, Av. Prat de la Riba 171, Edifici de Recerca (Gaudí), Santa Coloma de Gramenet, E08921 Barcelona, Spain

**Keywords:** tea, chicory, HPLC-UV, HPLC-FLD, fingerprinting, Chemometrics

## Abstract

Tea is a widely consumed drink in the world which is susceptible to undergoing adulterations to reduce manufacturing costs and rise financial benefits. The development of simple analytical methodologies to assess tea authenticity, as well as to detect and quantify frauds, is an important matter considering the rise of adulteration issues in recent years. In the present study, untargeted HPLC-UV and HPLC-FLD fingerprinting methods were employed to characterize, classify, and authenticate tea extracts belonging to different varieties (red, green, black, oolong, and white teas) by partial least squares-discriminant analysis (PLS-DA), as well as to detect and quantify adulteration frauds when chicory was used as the adulterant by partial least squares (PLS) regression, to ensure the authenticity and integrity of foodstuffs. Overall, PLS-DA showed a good classification and grouping of the tea samples according to the tea variety and, except for some white tea extracts, perfectly discriminated from the chicory ones. One hundred percent classification rates for the PLS-DA calibration models were achieved, except for green and oolong tea when HPLC-FLD fingerprints were employed, which showed classification rates of 96.43% and 95.45%, respectively. Good predictions were also accomplished, also showing, in almost all the cases, a 100% classification rate for prediction, with the exception of white tea and oolong tea when HPLC-UV fingerprints were employed that exhibited a classification rate of 77.78% and 88.89%, respectively. Good PLS results for chicory adulteration detection and quantitation were also accomplished, with calibration, cross-validation, and external validation errors beneath 1.4%, 6.4%, and 3.7%, respectively. Acceptable prediction errors (below 21.7%) were also observed, except for white tea extracts that showed higher errors which were attributed to the low sample variability available.

## 1. Introduction

Food adulteration and the absence of authenticity of beverages and foods have been increasing in the last years, being considered globally as one of the main food safety problems for consumers. In fact, food safety may be endangered by the use of fraudulent actions pursuing economic benefits, but that can represent important health problems for consumers when illegal (toxic) substances are employed or because of the presence of non-declared allergens. Food handling and adulteration practices are increasing because it is, in fact, easier to carry out fraud without being detected, and, today, it is one of the most important risks in food production which is gaining much attention from the industry and the governments, as well as from the standards-setting organizations [1]. In general, food adulteration is usually carried out to increase volume, mask the presence of inferior-quality components, or replace the authentic substances for the seller’s economic gain. The deliberate adulteration of foods and its misrepresentation to deceive the final consumers is illegal worldwide, and, in some cases, food fraud threats may be even riskier than traditional food safety issues since the contaminants are unconventional [2].

Tea is an aromatic drink made by pouring hot (or boiling) water over cured or fresh leaves of *Camellia sinensis*, an evergreen shrub original from China and East Asia. From there, it expanded to Europe by the (British) East India Company (founded in 1600) and the United (Dutch) East India Company (founded in 1602) [3]. There are mainly five types of tea, with green and black teas being the most traditional ones, representing around 22% and 78% of the production in the world, respectively [4]. Green tea is produced from dried harvested tea leaves followed by fermentation, while black tea is a fully oxidized product. Red tea (Pu-erh teas) is made with a specific variant of the tea plant (*Camellia sinensis var. assamica*) grown in the region of Yunnan (China), where dried tea leaves are subjected to composting under humid conditions favoring changes in the chemical composition caused by bacteria [5]. Oolong tea is also a fermented tea similar to the black one, but fermentation is controlled to limit oxidation between 10 to 70% [5]. White tea involves buds and younger tea leaves. It can also have been shaded from sunlight through its short growth to reduce the production of chlorophyll. It is dried soon after harvest to prevent fermentation, which needs to be carefully controlled [5]. 

The drinking of tea is also related to its well-recognized health properties, being proposed for the mitigation of minor diseases, such as headaches and pains, since their discovery back in China. In addition, tea extracts contain a wide variety of bioactive substances, among them polyphenols, being responsible for their antioxidant properties [5,6,7]. Evidence of anti-Alzheimer’s effect and prevention of obesity have also been described in tea extracts [8,9].

Together with coffee, tea is today one of the most worldwide consumed drinks, and it can be found among the food products that present more fraudulent practices. For example, tea is vulnerable to replacement with lower quality components, addition of undeclared flavors or colors, and mislabeling of geographical production origin and manufacture procedures. Tea shows a great variation in qualities when taken from different producers as they are grown under different environmental conditions of soil, rainfall, altitude, and methods of processing—especially fermentation—, but the extensive factor is the presence or absence of adulterants [10]. All these factors will influence the standard parameters which bring a variation in the market value of tea products. For instance, tea has been adulterated with different types of azo dyes, such as sunset yellow, tartrazine, and indigo carmine. In addition, starch, sand, China clay, French chalk, chicory, lather flakes, caffeine, and used tea leaves have also been employed as adulterants in tea products to reduce manufacturing costs and deceive the consumers [10,11]. 

Most of the strategies proposed for the detection of frauds are targeted methodologies, focusing on the determination and quantification of a specific analyte or group of analytes. Their concentrations or related signals are employed as food markers to assess their authenticity. For example, Ma et al. [12] studied the geographical production origin of several green teas from China by employing linear discriminant analysis based on the composition of 37 metal elements—including rare earth elements—quantified by inductively coupled plasma-mass spectrometry (ICP-MS). The determination of specific stable isotopes and elemental composition has also been described to assess the authenticity of teas [13,14,15,16,17,18]. Volatile profiling carried out by gas chromatography (GC) was also reported for the authentication of commercial powdered tea extracts [19]. In another application, Fang et al. evaluated the traceability of the production origin of Keemun black teas by employing its non-volatile composition, by means of the target analysis of phenolic compounds, caffeine, theanine, and theaflavins [20]. The use of commercially available standards (if quantitation is required) and the requirement of compound identification when profiling approaches are employed are among the main handicaps of targeted strategies, especially when considering the complexity of some food samples. In contrast, non-targeted methodologies (fingerprinting strategies) focus on detecting instrumental responses without any required knowledge about the food components [21]. With the idea of detecting as many components of the analyzed food matrices as possible, simple and unspecific sample treatments to avoid losing information are frequently employed [22], making non-targeted fingerprinting strategies the ideal methodologies for the authentication of tea products as they can be analyzed directly after pouring the tea with hot water. Near-infrared (NIR) [23,24,25,26,27,28], Fourier transform infrared (FTIR) [29], and ultraviolet-visible (UV-vis) [6,29,30] spectroscopies have been widely applied to generate fingerprints to address tea authentication issues. High-performance liquid chromatography with UV-detection (HPLC-UV) fingerprints have also been described for the authentication and discrimination of green tea samples [29], to authenticate Laoshan green tea plucking seasons [31], and to assess tea geographical origin [32], among other applications. Liquid chromatography coupled with mass spectrometry (LC-MS), particularly with high-resolution mass spectrometry (LC-HRMS), is also gaining popularity as a non-targeted fingerprinting strategy to address food authenticity, and some applications dealing with tea authentication issues have been reported. As an example, Navratilova et al. assessed the authentication of the geographic origin of Chinese, Japanese, and South Korean green teas by ultra-high performance liquid chromatography-high-resolution mass spectrometry (UHPLC-HRMS) metabolomic fingerprinting using a Synapt G2 MS system, which combined ion-mobility with HRMS in a quadrupole-time-of-flight (qTOF) analyzer [33]. The huge amount of chemical data provided by these techniques, especially when using non-targeted fingerprinting, will require the use of chemometric techniques to find relationships among chemical variables and sample features, as well as to assess the significance of the descriptors [34].

This research aims to study the application of untargeted high-performance liquid chromatography with ultraviolet (HPLC-UV) and fluorescence (HPLC-FLD) detection to characterize, classify, and authenticate tea samples based on their variety (black, green, red, oolong, and white teas). Under this fingerprinting strategy, conclusions can be observed without the requirement of identifying or quantifying the compounds responsible for the tea discrimination. With this objective, the HPLC-UV and HPLC-FLD chromatographic fingerprints obtained will be used as tea chemical descriptors. The resulting data will be submitted to chemometric multivariate methods, such as exploratory principal component analysis (PCA) and supervised partial least squares-discriminant analysis (PLS-DA), to study the distribution and classification of tea samples. Moreover, the feasibility of the developed chromatographic fingerprinting method for the detection of frauds and the quantitation of adulterated tea extracts with chicory, a plant root (*Chicorium intybus var. sativum*) which is employed as a non-declared tea ingredient on many occasions (i.e., an adulterant), will also be evaluated. Partial least square (PLS) regression will be employed to quantify chicory percentages in adulterated tea extracts.

## 2. Materials and Methods

### 2.1. Reagents and Chemicals

Methanol (Chromosolv™ for HPLC, ≥99.9%) was obtained from PanReac AppliChem (Barcelona, Spain) and formic acid (≥98%) from Sigma-Aldrich (St. Louis, MO, USA). Water was purified with an Elix 3 coupled to a Milli-Q system from Millipore Corporation (Millipore, Bedford, MA, USA) and was filtered through a 0.22 µm nylon membrane integrated into the Milli-Q system. A commercially available mineral water, purchased from Eroski (Barcelona, Spain) was employed for tea and chicory extraction to prevent any influence from the water in the obtained results. The chemical composition of this water was as follows: dry residue (at 180 °C): 403 mg/L; bicarbonate: 326 mg/L; chloride: 44 mg/L; calcium: 85 mg/L: magnesium: 28 mg/L; sodium: 18 mg/L; and silica: 8 mg/L.

### 2.2. Instrumentation

Non-targeted HPLC-UV and HPLC-FLD chromatographic fingerprints were obtained with an Agilent 1100 Series HPLC instrument (Waldbronn, Germany) equipped with an automatic injector, a binary pump, and both diode-array and fluorescence detectors (connected in series). Agilent Chemstation software was employed for instrument control and data processing. Reversed-phase separation using a Kinetex^®^ C18 (100 × 4.6 mm i.d., 2.6 µm partially porous particle size) column (Phenomenex, Torrance, CA, USA) was employed. Separation was carried out by gradient elution employing as mobile phase components a 0.1% formic acid aqueous solution and methanol. The gradient elution conditions are shown in Table 1. An injection volume of 5 µL was used. Acquisition was carried out at 280 nm for UV detection and at 280 nm (excitation) and 350 nm (emission) for FLD detection.

### 2.3. Samples and Sample Extraction Procedure

One hundred seven tea and chicory samples of different typologies (Table 2), and obtained from supermarkets in Barcelona (Spain) were used. Some sample characteristics, as the commercial name and production region, are listed in Table S1 (Supplementary Material).

The sample treatment consisted of extracting 0.5 g of tea/chicory sample with 25 mL of hot water in a 50 mL PTFE centrifuge tube (Serviquimia, Barcelona, Spain) by vigorously shaking for 1 min employing a Vortex (Stuart, Stone, United Kingdom). The obtained extracts were then centrifuged for 5 min at 3500 rpm (Rotanta 460 RS centrifuge, Hettich, Tuttlingen, Germany). The final aqueous extracts were filtered using 0.45 µm nylon filters (by discarding the first mL) and kept at 4 °C in amber glass injection vials until their analysis. 

Fifty microliters of each aqueous sample extract were mixed to prepare a quality control (QC) solution that was employed to study the repeatability of the proposed methods and the robustness of chemometric results.

### 2.4. Data Analysis

The sample aqueous extracts were randomly analyzed. After each ten sample extracts injected, an instrumental blank (mineral water) and a QC solution were analyzed. Data matrices were built with the obtained HPLC-UV or with the HPLC-FLD fingerprints of the analyzed teas and were subjected to principal component analysis (PCA), partial least squares-discriminant analysis (PLS-DA), and partial least squares (PLS) regression using SOLO 8.6 chemometric software from Eigenvector Research (Manson, WA, USA). The theoretical background of these chemometric methods are given elsewhere [35]. 

PCA was used as an exploratory methodology to study the distribution of samples and the QCs behavior. PLS-DA was used as a supervised classificatory method and PLS to quantify the chicory percentages added to the tea samples. In all cases, the X-data matrix was based on the registered HPLC-UV (absorbance versus retention time) or the registered HPLC-FLD (fluorescence intensity versus retention time) fingerprints. In contrast, Y-data matrices established each sample class and the adulterant percentage employed for PLS-DA and PLS, respectively. All the registered HPLC fingerprints were pretreated by smoothing, baseline-correcting, and aligning to improve the quality of the obtained chemometric data. Finally, data was autoscaled to achieve the same weight to each variable by minimizing differences in the magnitude and amplitude of their scales. PLS-DA model validation was carried out by using 70% of the samples (randomly selected) as the calibration set and the remaining 30% of the samples as the prediction set. The number of latent variables (LVs) in PLS-DA was obtained as the first important minimum point of the cross-validation (CV) error by a Venetian blind method. In the case of the PLS studies, adulteration cases of each tea sample class with chicory were evaluated, and the models were built and validated by using calibration and validation sets as defined in Table 3. 

The external prediction was also performed by means of adulterated samples using different tea and chicory samples than the ones used to made the PLS models at 15, 50, and 85% of chicory as an adulterant.

## 3. Results and Discussion

### 3.1. HPLC-UV and HPLC-FLD Fingerprints

**Figure 1:**
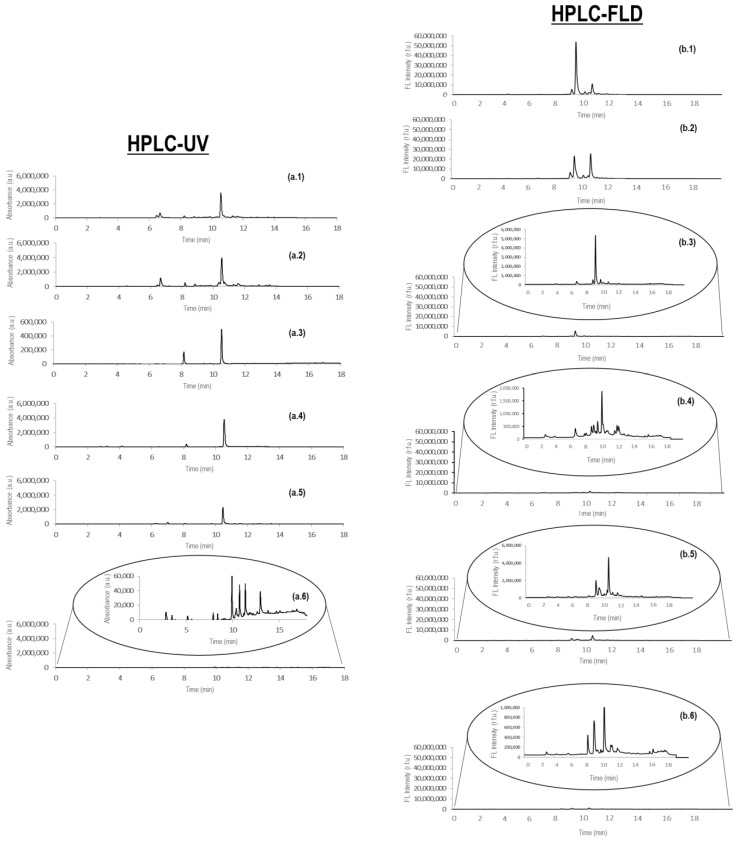
Non-targeted HPLC-UV (**a**) (at 280 nm) and HPLC-FLD (**b**) (at 280 nm (excitation) and 350 nm (emission)) fingerprints observed for a selected sample of each analyzed class: black (**a.1**,**b.1**), green (**a.2**,**b.2**), white (**a.3**,**b.3**), red (**a.4**,**b.4**), and oolong (**a.5**,**b.5**) teas, and chicory (**a.6**,**b.6**).

Figure 1a,b show, respectively, the obtained HPLC-UV and HPLC-FLD fingerprints for a selected sample of each analyzed class (black, green, oolong, red, and white teas, and chicory). 

Without taking into consideration the identity of the extracted and detected compounds, noteworthy differences can be detected among the chromatograms concerning the amount of peaks detected, their distribution, and the peak signals. In the case of HPLC-UV (Figure 1a.1–a.6), chicory samples provide a richer fingerprint in terms of detected peak signals in comparison to the ones obtained for tea samples, although peak signal intensities are 100-fold lower. Tea extracts tend to provide a similar HPLC-UV fingerprint. For example, an intense peak signal is observed at a retention time close to 11 min independently of the tea variety. In addition, more peak signals were observed for black and green tea samples in comparison to the other three tea varieties. Differences in peak signal intensities are also observed among the different tea classes, being white tea the one providing the most intense profiles. Similar behavior can also be observed when studying the obtained HPLC-FLD fingerprints (Figure 1b.1–b.6), although it seems that the chromatographic fingerprints are more different among the analyzed tea samples in comparison to the ones obtained with HPLC-UV. Again, the chicory sample showed the most different HPLC-FLD fingerprint. In this case, black and green teas are providing more intense signals in comparison with the other tea extracts, while, in contrast, red teas tend to provide richer fingerprints in terms of peaks detected, a comparable behavior with that observed for chicory samples.

The noteworthy differences detected by HPLC-UV and HPLC-FLD fingerprinting, and the fact that these observed characteristics were reproducible within the samples of each sample class (each specific tea and the chicory samples), suggested that the resulting data could be used as chemical descriptors to classify the studied samples by means of multivariate chemometric procedures. 

### 3.2. Sample Exploration by PCA

**Figure 2:**
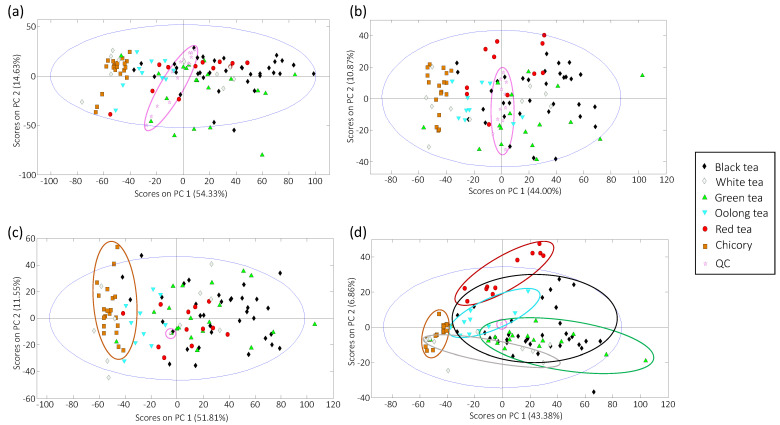
Principal component analysis (PCA) score plots of PC1 versus PC2 of the analyzed tea and chicory samples, and QCs. (**a**) Raw HPLC-UV fingerprints; (**b**) raw HPLC-FLD fingerprints; (**c**) HPLC-UV fingerprints after correction with QCs; (**d**) HPLC-FLD fingerprints corrected with QCs.

PCA was employed as an exploratory procedure to evaluate sample distribution according to the obtained fingerprinting features but mainly to assess the data repeatability and the robustness of the obtained chemometric results from the study of QCs. So, PCA score plots of PC1 versus PC2 using HPLC-UV fingerprints and HPLC-FLD fingerprints are shown, respectively, in Figure 2a,b. 

Working with the obtained raw fingerprinting data, it can be observed that QCs do not appear in a compact group, showing a dependence with the injection order in the employed analytical sequence, and this behavior is observed with both UV (Figure 2a) and FLD (Figure 2b) detection. This poor reproducibility may be related to changes in the lamps’ intensity during the sequence. Consequently, the distribution of the analyzed samples within the PCA score plot should be affected by the same problem; thus, a corrective mechanism should be applied to diminish the influence of the variations in sensitivity through the employed analytical series. This correction will rely on the QC injection performances. With this aim, the obtained HPLC-UV and HPLC-FLD fingerprints were corrected by dividing their signals with those obtained in the closest QC within the analysis sequence (each QC fingerprint was divided by itself). After this QC correction, all the QCs will be depicted in the same plot point (all the variables within their fingerprints will equal 1), whereas the variation in signal sensitivities caused by lamp fluctuations within the analysis sequence will be corrected. The plots of scores of PC1 versus PC2 from corrected data are depicted in Figure 2c,d, respectively. With this correction performed, samples tend to be better grouped based on their sample class, although this grouping was better achieved with HPLC-FLD fingerprints (Figure 2d). In any case, chicory samples are grouped at the left part of the plots, showing negative PC1 values, while tea samples are mainly located to the right side of the score plots, distinguished by increased values of PC1. Samples tend to be grouped in more compact groups with HPLC-FLD fingerprints (Figure 2d) in comparison to HPLC-UV counterparts (Figure 2c), and discrimination among the different tea types was also observed through PC2. For example, red and oolong teas exhibit positive PC2 values, while green and white teas tend to be distributed at the bottom area of the plot, with negative PC2 values. However, it should be mentioned that PCA is not a classificatory method, so, corrected HPLC-UV and HPLD-FLD fingerprints were further submitted to a PLS-DA classificatory method.

### 3.3. Sample Classification by Partial Least Squares-Discriminant Analysis

**Figure 3:**
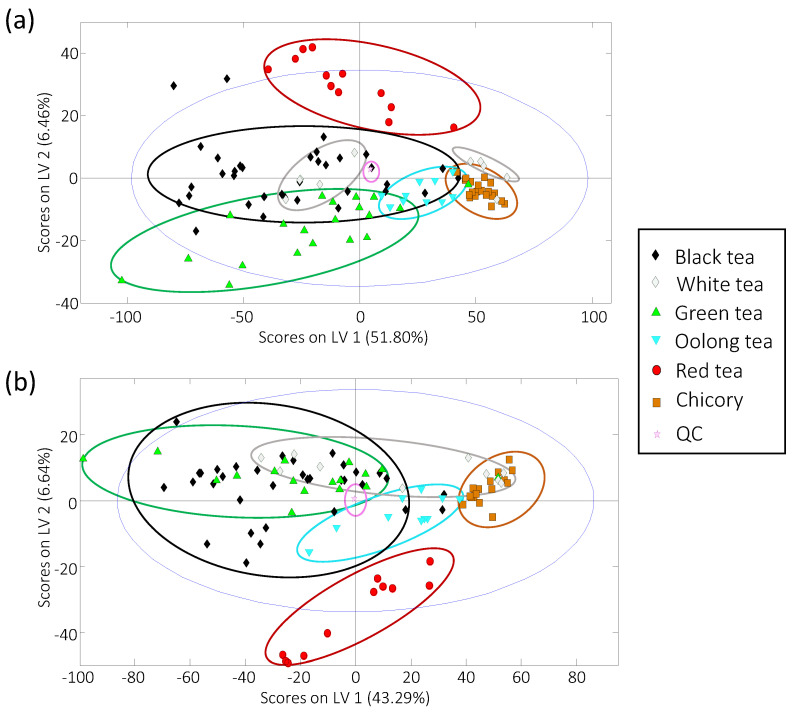
Score plots of LV1 versus LV2 of the analyzed tea and chicory samples by partial least squares-discriminant analysis (PLS-DA) when (**a**) HPLC-UV and (**b**) HPLC-FLD corrected fingerprints were used as chemical descriptors.

PLS-DA was employed to evaluate the characterization and classification of the analyzed tea and chicory samples. Figure 3a,b depict the plots of scores of LV1 versus LV2 using, respectively, HPLC-UV and HPLC-FLD corrected fingerprints as chemical descriptors. 

It can be highlighted that almost a perfect discrimination among chicory and tea extracts was obtained from both UV and FLD chromatograms, except for some white tea samples. This is a remarkable result as chicory is a low quality and low cost raw material used as a typical adulterant of teas. As can be seen in Figure 3, chicory samples are grouped in a compact area at the right of the plots, exhibiting positive values for LV1, regardless of the fingerprints employed. In contrast, perfect discrimination among the different studied tea extracts was not accomplished, although a different behavior can be observed depending on the type of fingerprints. For example, in the case of HPLC-UV (Figure 3a), red tea extracts are clearly differentiated and separated from the other teas based on LV2 (exhibiting positive values), while green tea samples are spread at the bottom of the PLS-DA plot, showing negative LV2 values. The other three types of tea under study are widely distributed through the LV1 axis. Two groups are observed for white samples, one of them located quite close to the chicory samples with some superposition. The clear discrimination of the white samples in these two groups can be attributed to the fact that all the samples came from different lots of two different white tea types, one produced in Fujian (China) and the other of a premium artisanal white tea production, also from China. Oolong tea samples were compactly grouped, although also close to the chicory samples. With HPLC-FLD fingerprints (Figure 3b), again, red tea samples were perfectly discriminated from the other tea groups, depicted at the bottom of the plot of scores. Similarly, oolong tea samples were grouped close to the chicory samples, although in a less compacted group in comparison with HPLC-UV fingerprints, and two groups were also found for white tea samples, one of them mixed with the chicory ones. No discrimination was observed between black and green tea samples, that appeared mixed in the left zone of the plot of scores. 

**Figure 4:**
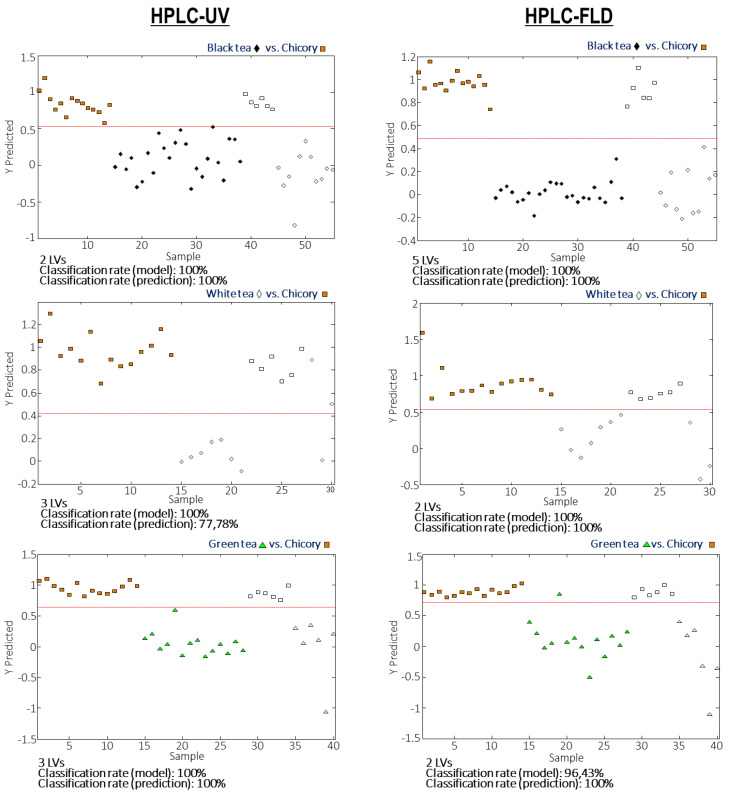

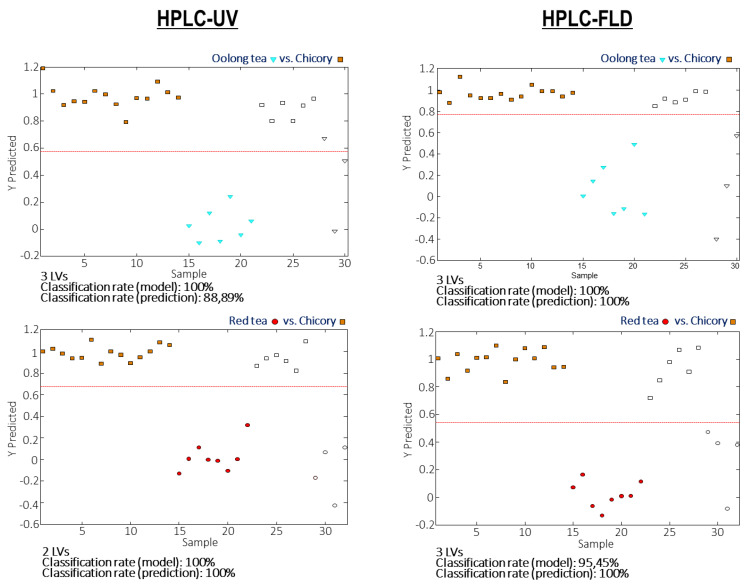
Validation of the paired PLS-DA models when using HPLC-UV and HPLC-FLD corrected chromatographic fingerprints as sample chemical descriptors. Red line establishes the separation between both classes.

Even though full discrimination among the analyzed tea samples was not accomplished, most of the tea extracts, except for some white tea samples, are differentiated from the chicory ones, as previously commented. This finding allows for proposing the HPLC-UV and HPLC-FLD fingerprints as good chemical descriptors to guarantee tea authentication when frauds based on chicory adulterations are produced. To ensure the application of the developed methodology for fraud detection, the classification rate of tea samples in front of chicory ones was studied from paired PLS-DA models (each tea variety versus chicory). For that purpose, paired PLS-DA models were built by using 70% of the samples (randomly selected) for each pair tea-chicory as the calibration set, and the remaining 30% of the samples were considered as “unknown samples” for validation and prediction purposes. Figure 4 depicts the validation results of the paired PLS-DA models when HPLC-UV and HPLC-FLD corrected chromatographic fingerprints were used as chemical descriptors. The obtained PLS model and prediction classification rates are also summarized in Table S2 (Supplementary Material).

Very acceptable results were achieved. One hundred percent classification rates for the calibration models were obtained in all the cases except for green and oolong tea using HPLC-FLD data, which showed a classification rate of 96.43% and 95.45%, respectively. A good prediction was also accomplished, with a 100% classification rate for prediction in almost all the cases, except for white tea and oolong tea, when HPLC-UV fingerprints were employed that exhibited a classification rate of 77.78% and 88.89%, respectively. These results show that, a priori, both HPLC-UV and HPLC-FLD corrected chromatographic fingerprints can be proposed as good sample chemical descriptors to address tea extract authentication when adulterated with chicory samples.

### 3.4. Detection and Quantitation of Tea Adulterations with Chicory by PLS

**Figure 5:**
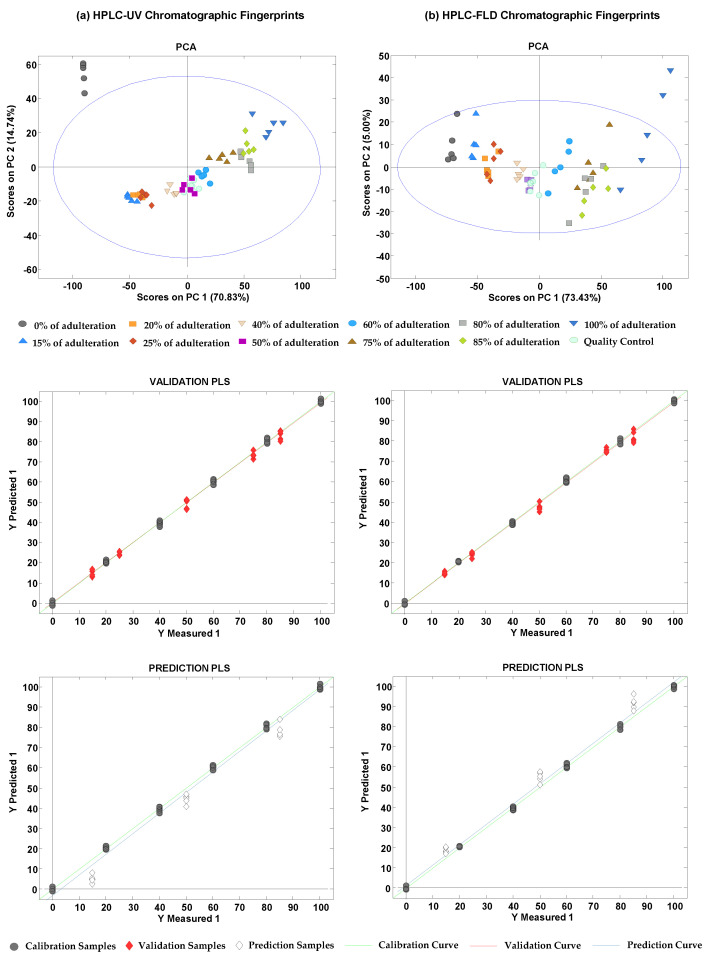
PCA plots depicting the distribution of both calibration and validation sets, and the scatter plots of measured versus predicted percentages of adulterant (chicory) by PLS when a red tea sample was adulterated with chicory for external validation, by employing HPLC-UV (**a**) and HPLC-FLD (**b**) corrected fingerprints.

Five adulteration cases were studied to evaluate the viability of the proposed HPLC-UV and HPLC-FLD corrected chromatographic fingerprints for the detection and quantitation of chicory adulterations in tea extracts. The adulteration cases under study involved each one of the tea groups (red, green, black, oolong, and white teas) which were adulterated with chicory at different percentages, all of them by quintuplicate (see Table 3). HPLC-UV and HPLC-FLD data were then subjected to PCA to explore sample distribution according to the percentage of adulterant, as well as to PLS to quantify the adulteration percentage. As an example, Figure 5 depicts the PCA plots showing the distribution of both calibration and validation samples, and the scatter plots of measured versus predicted percentage levels of adulteration (chicory) for a red tea sample adulterated with chicory, using HPLC-UV (Figure 5a) and HPLC-FLD (Figure 5b) corrected data. 

As can be seen, scatter plots of PCA scores distributed the adulterated samples according to the adulterant percentage, from pure red tea extracts (at the left of the plots) to pure chicory extracts (at the right of the plots). Similar performance was also achieved for the other cases under evaluation. In this adulteration study (red tea versus chicory), very good validation and prediction PLS results were obtained, regardless of the type of fingerprints, with linearities higher than 0.999, although, as expected, prediction errors of validation and prediction steps were higher taking into consideration that different sample extracts were used to build the calibration model. Table 4 summarizes the LVs used, the linearity (R^2^) and the PLS calibration, cross-validation, and external validation and prediction errors for all the adulteration cases under study.

As can be seen, similar figures of merit were achieved for both HPLC-UV and HPLC-FLD fingerprints. In all cases, good linearity values (R^2^ > 0.998) and calibration errors and cross-validation errors lower than 1.4% and 6.4%, respectively, were achieved. As commented in Section 2.4, PLS external validation was carried out at different chicory adulteration levels using the same tea and chicory extracts for building the PLS models. As shown in the table, quite acceptable external validation errors, with values below 2.2% and 3.7% for HPLC-UV and HPLC-FLD fingerprints, respectively, were obtained. In contrast, the prediction step relied on different tea and chicory extracts than the ones employed for building the PLS models. As expected, an increase in the prediction errors was observed, showing a different behavior depending on the tea variety. For most of the adulteration cases under study, prediction errors increased up to 21.6% and 18.3% for HPLC-UV and HPLC-FLD, respectively. Better predictions were obtained using HPLC-FLD fingerprints for red tea and black tea adulterated with chicory, with prediction errors of 5.7% and 2.6%, respectively. The contribution in the increment on the prediction errors observed came mainly for the prediction of the lowest (15%) and the highest (85%) adulteration levels, as can be seen in the prediction PLS plots of Figure 5. Nevertheless, these values can be considered acceptable taking into consideration the type of application under study, as tea adulteration with low chicory levels will not be expected (no economic benefit), but either high chicory adulteration levels are expected to be employed (the adulteration may be then easily detected by visual inspection). Only in the case of white tea extracts adulterated with chicory did none of the proposed HPLC-UV and HPLC-FLD methods seem to be useful for predicting adulteration levels when different sample extracts than the ones used for building the PLS calibration models, showing very high prediction errors (80.7% and 132.9% for HPLC-UV and HPLC-FLD fingerprints, respectively). This finding was attributed to the similarity in the compositional fingerprints of white teas and chicory samples as they were always in close locations in the plots of scores of the previous PCA and PLS-DA systems.

## 4. Conclusions

Untargeted HPLV-UV fingerprints acquired at 280 nm and HPLC-FLD fingerprints acquired at 280 nm and 350 nm for excitation and emission, respectively, have shown to be appropriate chemical descriptors to characterize, classify, and authenticate tea samples belonging to the main five tea varieties (red, green, black, oolong, and white teas). Good discrimination of the tea samples against chicory extracts was achieved by PLS-DA. In addition, 100% classification rates (in both, calibration and prediction evaluation) were achieved when validating paired PLS-DA models of each tea extract type against chicory, with very few exceptions, thus enhancing the classification and authentication capacity of the developed methodology.

PLS was employed in several adulteration cases representing each tea extract variety adulterated with chicory. Generally, good PLS results for chicory adulteration detection and quantitation were also accomplished, with calibration, cross-validation, and external validation errors below 1.4%, 6.4%, and 3.7%, respectively. Acceptable prediction errors using adulterated samples prepared with different extracts than the ones employed for PLS calibration (below 21.7%) were also observed, except for the white tea extracts that showed higher errors, a fact that was attributed to the similarity of their fingerprints.

The proposed non-targeted HPLC-UV and HPLC-FLD fingerprinting methods can be used as a simple and inexpensive approach to ensure and guarantee tea extract authenticity, as well as to prevent fraudulent practices against adulteration with chicory (a common non-tea-based adulterant). 

## Figures and Tables

**Table 1:** Gradient elution conditions.

Time (min)	Elution	% Methanol	Flow Rate (µL/min)
0–15	Linear gradient	20–75%	400
15–17	Linear gradient	75–95%	400
17–19	Isocratic	95%	400
19–19.2	Linear gradient	95–20%	400
19.2–25	Isocratic	20%	400

**Table 2:** Summary of the studied samples.

Sample Class	Sample Type	Number of Samples
Tea	Black tea	35
	Green tea	20
	Oolong tea	10
	Red tea	12
	White tea	10
Chicory	Chicory	20

**Table 3:** Samples employed for calibration and validation in the adulteration studies by PLS (*n* = 5 for each sample).

	Tea (%)	Chicory (% Adulterant)
Calibration set	100	0
	80	20
	60	40
	40	60
	20	80
	0	100
Validation set	85	15
	75	25
	50	50
	25	75
	15	85

**Table 4:** Results for the evaluation of adulteration of tea samples with chicory using HPLC-UV and HPLC-FLD as chemical descriptors for PLS.

	**HPLC-UV Fingerprints**					
	**LVs**	**Linearity (R^2^)**	**Calibration Error** **(%)**	**Cross-Validation Error (%)**	**External Validation** **Error (%)**	**Prediction Error (%)**
Red tea	4	0.999	1.0	1.5	2.0	8.0
Green tea	5	0.999	0.5	1.3	1.0	15.7
Black tea	4	0.999	1.0	3.6	2.1	21.6
Oolong tea	2	0.998	1.4	6.4	1.8	15.0
White tea	3	0.999	1.3	2.3	2.2	80.7
	**HPLC-FLD Fingerprints**					
	**LVs**	**Linearity (R^2^)**	**Calibration Error (%)**	**Cross-Validation** **Error (%)**	**External Validation** **Error (%)**	**Prediction Error (%)**
Red tea	4	0.999	0.9	2.4	2,5	5.7
Green tea	5	0.999	0.3	1.1	1.4	18.3
Black tea	3	0.999	1.1	1.6	1.6	2.6
Oolong tea	2	0.998	1.4	1.9	1.4	13.0
White tea	3	0.999	0.8	2.3	3.7	132.9

## Data Availability

Data is available upon request to the authors.

## Supplementary Materials

The following are available online at https://www.mdpi.com/article/10.3390/foods10122935/s1, Table S1: Information about the analyzed samples, Table S2: PLS model and prediction classification rates in the tea extract versus chicory adulteration studies.
