# Supplementary material for: Tea and Chicory Extract Characterization, Classification and Authentication by Non-Targeted HPLC-UV-FLD Fingerprinting and Chemometrics
Source: Foods. 2021 Nov 28;10(12):2935. doi: 10.3390/foods10122935 (PMC8700607; doi:10.3390/foods10122935)
Supplement: Supplementary file 1 [file foods-10-02935-s001.zip › foods-1470625-supplementary.pdf]

Supplementary Material

Tea and Chicory Extract Characterization, Classification and Authentication by non-Targeted HPLC-UV-FLD Fingerprinting and Chemometrics

Josep Pons [1], Àlex Bedmar [1], Nerea Núñez [1], Javier Saurina [1,2] and Oscar Núñez [1,2,*]

[1] Department of Chemical Engineering and Analytical Chemistry, University of Barcelona. Martí i Franquès 1-11, E08028, Barcelona, Spain. (J.P., josepferre8@gmail.com, À.B., alexbedmar1999@gmail.com, N.N., nereant7@gmail.com, J.S., xavi.saurina@ub.edu; O.N., oscar.nunez@ub.edu)

[2] Research Institute in Food Nutrition and Food Safety, University of Barcelona, Recinte Torribera, Av. Prat de la Riba 171, Edifici de Recerca (Gaudí), Santa Coloma de Gramenet, E08921 Barcelona, Spain.

* Correspondence: O.N.: oscar.nunez@ub.edu

Table S1. Information about the analyzed samples.

Sample type	Commercial name	Production region	Number of samples (different lots)
Black tea	Ceylon black tea	Sri Lanka	3
	Irish blend	India	3
	Strong English breakfast	Sri Lanka	3
	Yunnan finest tippy, premium	China	2
	India Assam maud	India	3
	Kenya Marnyin	Kenya	3
	Darjeeling ringtong (second harvest)	India	3
	Darjeeling first flush (first harvest)	India	2
	Formosa tarry lapsang souchong	Taiwan	3
	Ceylon quality blend	Sri Lanka	2
	Ceylon Nuwara eliya	Sri Lanka	3
	Korakundah mountain tea	India	3
	Darjelling margaret's hope (first harvest, premium)	India	2
Green tea	Organic gunpowder	China	2
	Pi lo chun (premium)	Taiwan	2
	Sencha (Zhejiang)	China	2
	Lung ching	China	2
	Sencha (premium)	China	2
	Japan Bancha premium	Japan	2
	Japan gyokuro organic	Japan	2
	Mao Feng Jiangsu	China	2
	Assam Jamguri green	India	2
	Lung ching second grade premium	China	2
Oolong tea	Dong ding oolong	Taiwan	3

	Tie kuan yin	China	3
	Milky oolong	China	2
	Special yellow sun	China	2
Red tea	Pu erh Royal (special fermentation)	China	3
	Pu erh Original	China	3
	Pu erh Imperial (manual harvesting)	China	3
	Pu erh Royal Palace	China	3
White tea	Pai Mu tan	China	5
	Silver needles (premium, artisanal production)	China	5
Chicory	Chicory roots, Valley of Tea	Belgium	5
	Ecologial chicory, Herbes del Molí	Spain	5
	Chicory roots, Health Embassy	England	5
	Chicory roots, Especias Pedroza	Spain	5

Table S2. PLS model and prediction classification rates in the tea extract vs. chicory adulteration studies.

Adulteration study	HPLC-UV fingerprint		HPLC-FLD fingerprint	
	**PLS model**	**PLS prediction**	**PLS model**	**PLS prediction**
Black tea vs. chicory	100%	100%	100%	100%
White tea vs. chicory	100%	77.78%	100%	100%
Green tea vs. chicory	100%	100%	96.43%	100%
Oolong tea vs. chicory	100%	88.89%	100%	100%
Red tea vs. chicory	100%	100%	94.45%	100%
